# Crystal structure of 2-bromo-3-di­methyl­amino-*N*,*N*,*N*′,*N*′,4-penta­methyl-4-(tri­methyl­sil­yloxy)pent-2-eneamidinium bromide

**DOI:** 10.1107/S205698901502383X

**Published:** 2015-12-16

**Authors:** Ioannis Tiritiris, Ralf Kress, Willi Kantlehner

**Affiliations:** a Fakultät Chemie/Organische Chemie, Hochschule Aalen, Beethovenstrasse 1, D-73430 Aalen, Germany

**Keywords:** crystal structure, bromide, amidinium, salt, Br⋯Br inter­actions, C—H⋯Br inter­actions

## Abstract

The reaction of the ortho­amide 1,1,1-tris­(di­methyl­amino)-4-methyl-4-(tri­methyl­sil­yloxy)pent-2-yne with bromine in benzene, yields the title salt, C_15_H_33_BrN_3_OSi^+^·Br^−^. The C—N bond lengths in the amidinium unit are 1.319 (6) and 1.333 (6) Å, indicating double-bond character, pointing towards charge delocalization within the NCN plane. The C—Br bond length of 1.926 (5) Å is characteristic for a C—Br single bond. Additionally, there is a bromine–bromine inter­action [3.229 (3) Å] present involving the anion and cation. In the crystal, weak C—H⋯Br inter­actions between the methyl H atoms of the cation and the bromide ions are present.

## Related literature   

For the nature of halogen–halogen inter­actions in crystals, see: Desiraju & Parthasarathy (1989 ▸). For the synthesis of alkynyl ortho­amides and propiolamidinium salts, see: Weingärtner *et al.* (2011 ▸). For the synthesis of vinyl­ogous guanidinium iodides and bromides, see: Kantlehner *et al.* (2012*a* ▸). For the crystal structure of 3-phenyl-*N*,*N*,*N*′,*N*′′-tetra­methyl-1-ethyne-1-carboximidamidium bromide, see: Tiritiris & Kantlehner (2012*b* ▸).
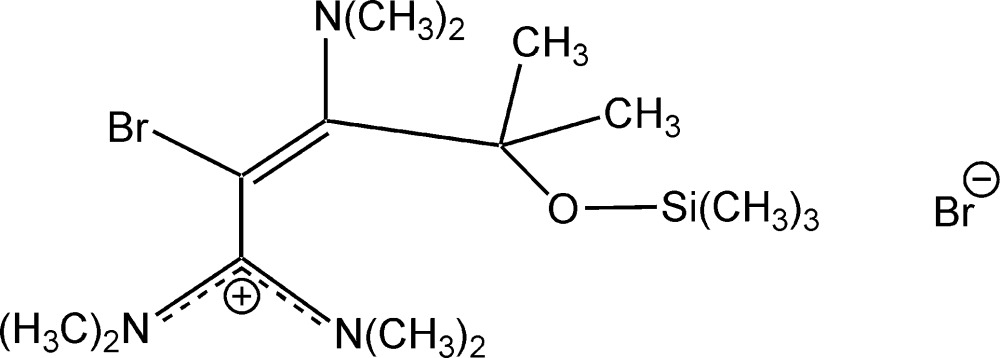


## Experimental   

### Crystal data  

C_15_H_33_BrN_3_OSi^+^·Br^−^
*M* *_r_* = 459.33
Orthorhombic, 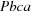

*a* = 13.3524 (5) Å
*b* = 11.3802 (3) Å
*c* = 27.4261 (14) Å
*V* = 4167.5 (3) Å^3^
*Z* = 8
Mo *K*α radiation
μ = 3.95 mm^−1^
*T* = 100 K
0.45 × 0.30 × 0.15 mm

### Data collection  

Bruker Kappa APEXII DUO diffractometer
Absorption correction: multi-scan (Blessing, 1995 ▸) *T*_min_ = 0.285, *T*_max_ = 0.530
32287 measured reflections
5164 independent reflections
3394 reflections with *I* > 2σ(*I*)
*R*_int_ = 0.095

### Refinement  

*R*[*F* ^2^ > 2σ(*F* ^2^)] = 0.061
*wR*(*F* ^2^) = 0.099
*S* = 1.17
5164 reflections
210 parameters
H-atom parameters constrained
Δρ_max_ = 1.10 e Å^−3^
Δρ_min_ = −1.88 e Å^−3^

### 

Data collection: *APEX2* (Bruker, 2008 ▸); cell refinement: *SAINT* (Bruker, 2008 ▸); data reduction: *SAINT*; program(s) used to solve structure: *SHELXS97* (Sheldrick, 2008 ▸); program(s) used to refine structure: *SHELXL2014* (Sheldrick, 2015 ▸); molecular graphics: *DIAMOND* (Brandenburg & Putz, 2005 ▸); software used to prepare material for publication: *SHELXL2014*.

## Supplementary Material

Crystal structure: contains datablock(s) I, global. DOI: 10.1107/S205698901502383X/ff2146sup1.cif

**Fig. 1.**
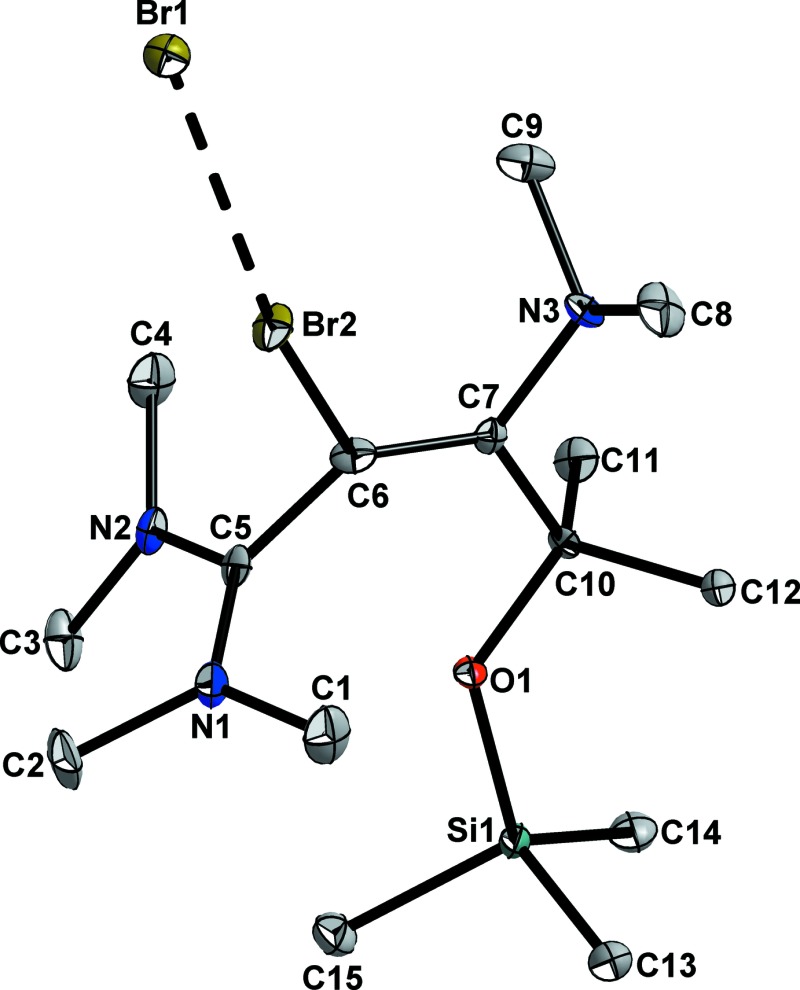
The structure of the title compound with displacement ellipsoids at the 50% probability level. All hydrogen atoms were omitted for the sake of clarity. The Br···Br interaction is indicated by a black dashed line.

. DOI: 10.1107/S205698901502383X/ff2146fig1.tif
The structure of the title compound with displacement ellipsoids at the 50% probability level. All hydrogen atoms were omitted for the sake of clarity. The Br⋯Br inter­action is indicated by a black dashed line.

**Fig. 2.**
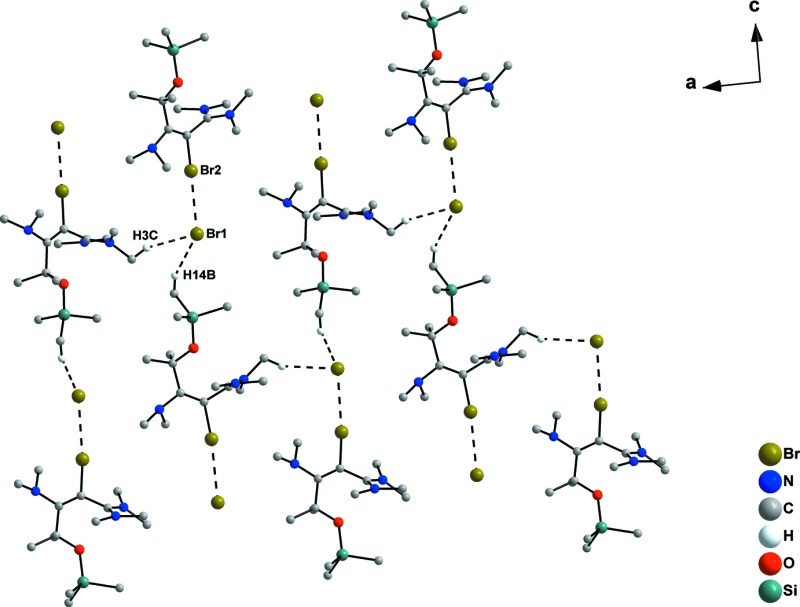
C—H···Br interactions (black dashed lines) between the hydrogen atoms of the methyl groups and the bromide ions. Br···Br interactions are also indicated by black dashed lines.

. DOI: 10.1107/S205698901502383X/ff2146fig2.tif
C—H⋯Br inter­actions (black dashed lines) between the hydrogen atoms of the methyl groups and the bromide ions. Br⋯Br inter­actions are also indicated by black dashed lines.

CCDC reference: 1441961

Additional supporting information:  crystallographic information; 3D view; checkCIF report

## Figures and Tables

**Table 1:** Hydrogen-bond geometry (Å, °)

*D*—H⋯*A*	*D*—H	H⋯*A*	*D*⋯*A*	*D*—H⋯*A*
C3—H3*C*⋯Br1^i^	0.98	2.81	3.742 (3)	159
C14—H14*B*⋯Br1^ii^	0.98	2.87	3.790 (3)	156

Symmetry codes: (i) 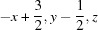; (ii) 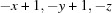.

## supplementary crystallographic information

### S1. Comment

Orthoamide derivatives of alkynecarboxylic acids are prepared from *N*,*N*,*N*',*N*',*N''*,*N''*- hexaalkylguanidinium chlorides and terminal alkynes. Their conversion into propiolamidinium chlorides by reaction with benzoyl chloride and into propiolamidinium trifluoromethanesulfonates by reaction with triethylsilyl trifluoromethanesulfonate is well known in literature (Weingärtner *et al.*, 2011). Alkyne orthoamides are transformed by elemental iodine or bromine to vinylogous guanidinium iodides or bromides (Kantlehner *et al.*, 2012*a*). Phenyl substituted alkyne orthoamides like 3,3,3-Tris(dimethylamino)-1-phenyl- prop-1-yne (Weingärtner *et al.*, 2011) behave differently, it reacts with bromine to give 3-Phenyl-*N*,*N*,*N*',*N''*- tetramethyl-1-ethyne-1-carboximidamidium bromide (Tiritiris & Kantlehner, 2012*b*). According to the structure analysis of the title compound, the C–N bond lengths in the amidinium unit are 1.319 (6) and 1.333 (6) Å, indicating double bond character. The positive charge in the cation is distributed between both dimethylamino groups. The bromine atom Br2 and the 3-dimethylamino group are in *cis* position due to sterical reasons (Fig. 1). The angle between the planes N1/C5/N2 and C10/C7/N3 is 85.1 (1)°. Other prominent bond parameters in the cation are: C6–Br2 = 1.926 (5) Å and C6–C7 = 1.327 (7) Å, characteristic for a C–Br single and C–C double bond, respectively. Additionally, an bromine-bromine interaction [*d*(Br···Br) = 3.229 (3) Å] between the anion and cation has been determined, which is shorter than the sum of their van der Waals radii (Desiraju & Parthasarathy, 1989). Week C–H···Br interactions between the hydrogen atoms of –N(CH_3_)_2_ and –SiCH_3_ groups and the bromide ions are present (Fig. 2), ranging from 2.81 to 2.87 Å (Tab. 1). Typical values of Br···Br, C···Br and H···Br interactions in bromohydrocarbon crystals were considered by Desiraju and Parthasarathy having less than 3.72, 3.61 and 3.06 Å, respectively (Desiraju & Parthasarathy, 1989).

### S2. Experimental

To (2.10 g, 7.0 mmol) 4-methyl-4-trimethylsilyloxy-1,1,1- tris(dimethylamino)pent-2-yne in 50 ml benzene was added dropwise under ice/water cooling, elemental bromine (1.12 g, 7.0 mmol) in benzene. After stirring for two hours at room temperature, a yellow precipitate was collected by filtration. The title compound crystallized from a saturated acetonitrile solution after several days at 273 K, forming yellow single crystals. Yield: 2.77 g (80%).

### S3. Refinement

The hydrogen atoms of the methyl groups were allowed to rotate with a fixed angle around the C–N, C–C and C–Si bonds to best fit the experimental electron density, with *U*_iso_(H) set to 1.5 *U*_eq_(C) and d(C—H) = 0.98 Å.

### Crystal data

**Table:**

C_15_H_33_BrN_3_OSi^+^·Br^−^	*D*_x_ = 1.464 Mg m^−^^3^
*M**_r_* = 459.33	Mo *K*α radiation, λ = 0.71073 Å
Orthorhombic, *P**b**c**a*	Cell parameters from 32287 reflections
*a* = 13.3524 (5) Å	θ = 1.5–28.3°
*b* = 11.3802 (3) Å	µ = 3.95 mm^−^^1^
*c* = 27.4261 (14) Å	*T* = 100 K
*V* = 4167.5 (3) Å^3^	Block, yellow
*Z* = 8	0.45 × 0.30 × 0.15 mm
*F*(000) = 1888	

### Data collection

**Table:**

Bruker Kappa APEXII DUO diffractometer	5164 independent reflections
Radiation source: fine-focus sealed tube	3394 reflections with *I* > 2σ(*I*)
Triumph monochromator	*R*_int_ = 0.095
φ scans, and ω scans	θ_max_ = 28.3°, θ_min_ = 1.5°
Absorption correction: multi-scan (Blessing, 1995)	*h* = −17→10
*T*_min_ = 0.285, *T*_max_ = 0.530	*k* = −15→15
32287 measured reflections	*l* = −36→36

### Refinement

**Table:**

Refinement on *F*^2^	Primary atom site location: structure-invariant direct methods
Least-squares matrix: full	Secondary atom site location: difference Fourier map
*R*[*F*^2^ > 2σ(*F*^2^)] = 0.061	Hydrogen site location: inferred from neighbouring sites
*wR*(*F*^2^) = 0.099	H-atom parameters constrained
*S* = 1.17	*w* = 1/[σ^2^(*F*_o_^2^) + 19.9082*P*] where *P* = (*F*_o_^2^ + 2*F*_c_^2^)/3
5164 reflections	(Δ/σ)_max_ < 0.001
210 parameters	Δρ_max_ = 1.10 e Å^−^^3^
0 restraints	Δρ_min_ = −1.88 e Å^−^^3^

### Special details

**Table:**

Geometry. All e.s.d.'s (except the e.s.d. in the dihedral angle between two l.s. planes) are estimated using the full covariance matrix. The cell e.s.d.'s are taken into account individually in the estimation of e.s.d.'s in distances, angles and torsion angles; correlations between e.s.d.'s in cell parameters are only used when they are defined by crystal symmetry. An approximate (isotropic) treatment of cell e.s.d.'s is used for estimating e.s.d.'s involving l.s. planes.
Refinement. Refinement of *F*^2^ against ALL reflections. The weighted *R*-factor *wR* and goodness of fit *S* are based on *F*^2^, conventional *R*-factors *R* are based on *F*, with *F* set to zero for negative *F*^2^. The threshold expression of *F*^2^ > σ(*F*^2^) is used only for calculating *R*-factors(gt) *etc*. and is not relevant to the choice of reflections for refinement. *R*-factors based on *F*^2^ are statistically about twice as large as those based on *F*, and *R*- factors based on ALL data will be even larger.

### Fractional atomic coordinates and isotropic or equivalent isotropic displacement parameters (Å^2^)

**Table:**

	*x*	*y*	*z*	*U*_iso_*/*U*_eq_	
Br1	0.54519 (4)	0.73694 (4)	0.13843 (2)	0.01487 (12)	
Br2	0.45103 (4)	0.23306 (5)	0.24383 (2)	0.01507 (12)	
N1	0.5406 (3)	0.3518 (3)	0.13946 (16)	0.0147 (9)	
C1	0.4511 (4)	0.4276 (4)	0.1374 (2)	0.0213 (12)	
H1A	0.3938	0.3816	0.1258	0.032*	
H1B	0.4633	0.4930	0.1149	0.032*	
H1C	0.4367	0.4585	0.1700	0.032*	
C2	0.6368 (4)	0.4157 (5)	0.1374 (2)	0.0226 (13)	
H2A	0.6879	0.3714	0.1553	0.034*	
H2B	0.6287	0.4935	0.1521	0.034*	
H2C	0.6576	0.4244	0.1033	0.034*	
N2	0.6052 (3)	0.1612 (4)	0.14393 (15)	0.0125 (9)	
C3	0.6869 (4)	0.1740 (5)	0.1081 (2)	0.0183 (12)	
H3A	0.6711	0.2385	0.0857	0.027*	
H3B	0.6942	0.1008	0.0896	0.027*	
H3C	0.7496	0.1914	0.1252	0.027*	
C4	0.6060 (4)	0.0462 (5)	0.1683 (2)	0.0194 (12)	
H4A	0.5545	0.0448	0.1938	0.029*	
H4B	0.6719	0.0328	0.1831	0.029*	
H4C	0.5922	−0.0157	0.1444	0.029*	
C5	0.5318 (3)	0.2392 (4)	0.15005 (16)	0.0109 (10)	
C6	0.4400 (4)	0.1967 (4)	0.17545 (17)	0.0113 (10)	
C7	0.3621 (3)	0.1384 (4)	0.15758 (18)	0.0090 (10)	
N3	0.2856 (3)	0.0847 (4)	0.18667 (15)	0.0135 (10)	
C8	0.2094 (4)	0.1616 (5)	0.2075 (2)	0.0202 (13)	
H8A	0.2364	0.2005	0.2365	0.030*	
H8B	0.1506	0.1149	0.2166	0.030*	
H8C	0.1900	0.2209	0.1834	0.030*	
C9	0.3192 (4)	−0.0079 (5)	0.2199 (2)	0.0206 (13)	
H9A	0.3710	−0.0551	0.2039	0.031*	
H9B	0.2623	−0.0583	0.2284	0.031*	
H9C	0.3466	0.0277	0.2496	0.031*	
C10	0.3455 (4)	0.1153 (4)	0.10276 (18)	0.0087 (10)	
C11	0.3601 (4)	−0.0161 (4)	0.09338 (19)	0.0145 (11)	
H11A	0.3426	−0.0341	0.0595	0.022*	
H11B	0.3168	−0.0612	0.1154	0.022*	
H11C	0.4302	−0.0372	0.0993	0.022*	
C12	0.2409 (3)	0.1539 (4)	0.08853 (18)	0.0112 (10)	
H12A	0.2334	0.2383	0.0947	0.017*	
H12B	0.1916	0.1103	0.1078	0.017*	
H12C	0.2301	0.1381	0.0538	0.017*	
O1	0.4196 (2)	0.1804 (3)	0.07547 (12)	0.0096 (7)	
Si1	0.41809 (10)	0.21877 (12)	0.01674 (5)	0.0103 (3)	
C13	0.3405 (4)	0.3517 (5)	0.0054 (2)	0.0169 (12)	
H13A	0.2698	0.3332	0.0113	0.025*	
H13B	0.3491	0.3772	−0.0285	0.025*	
H13C	0.3616	0.4149	0.0274	0.025*	
C14	0.3757 (4)	0.0988 (5)	−0.02454 (19)	0.0170 (12)	
H14A	0.4182	0.0295	−0.0198	0.025*	
H14B	0.3804	0.1251	−0.0585	0.025*	
H14C	0.3060	0.0785	−0.0170	0.025*	
C15	0.5486 (4)	0.2599 (5)	0.00131 (18)	0.0196 (11)	
H15A	0.5687	0.3283	0.0207	0.029*	
H15B	0.5529	0.2791	−0.0335	0.029*	
H15C	0.5934	0.1939	0.0086	0.029*	

### Atomic displacement parameters (Å^2^)

**Table:**

	*U*^11^	*U*^22^	*U*^33^	*U*^12^	*U*^13^	*U*^23^
Br1	0.0172 (2)	0.0146 (3)	0.0128 (3)	−0.0002 (2)	0.0014 (2)	0.0024 (2)
Br2	0.0157 (2)	0.0198 (3)	0.0097 (2)	0.0007 (2)	−0.0020 (2)	−0.0043 (2)
N1	0.010 (2)	0.015 (2)	0.019 (2)	−0.0006 (18)	−0.003 (2)	−0.0009 (18)
C1	0.017 (3)	0.014 (3)	0.033 (3)	0.006 (2)	−0.003 (3)	−0.002 (2)
C2	0.013 (3)	0.018 (3)	0.036 (4)	−0.009 (2)	−0.004 (3)	−0.003 (3)
N2	0.009 (2)	0.013 (2)	0.015 (2)	0.0023 (16)	−0.0033 (18)	−0.0027 (18)
C3	0.011 (3)	0.024 (3)	0.020 (3)	0.002 (2)	0.002 (2)	−0.009 (3)
C4	0.017 (3)	0.014 (3)	0.027 (3)	0.005 (2)	−0.002 (2)	−0.003 (2)
C5	0.011 (2)	0.013 (2)	0.009 (2)	−0.002 (2)	−0.0038 (18)	−0.0037 (19)
C6	0.017 (3)	0.012 (2)	0.005 (2)	0.001 (2)	0.002 (2)	−0.0028 (19)
C7	0.009 (2)	0.008 (2)	0.011 (3)	0.0018 (18)	0.000 (2)	−0.0007 (19)
N3	0.011 (2)	0.017 (2)	0.013 (2)	−0.0023 (17)	0.0040 (17)	0.0027 (18)
C8	0.014 (3)	0.029 (3)	0.017 (3)	0.001 (2)	0.008 (2)	−0.004 (2)
C9	0.026 (3)	0.017 (3)	0.019 (3)	−0.001 (2)	0.005 (2)	0.005 (2)
C10	0.011 (2)	0.004 (2)	0.011 (3)	−0.0030 (18)	−0.002 (2)	0.000 (2)
C11	0.018 (3)	0.013 (3)	0.013 (3)	−0.001 (2)	−0.001 (2)	−0.004 (2)
C12	0.009 (2)	0.013 (3)	0.012 (3)	−0.0002 (19)	0.000 (2)	0.001 (2)
O1	0.0090 (16)	0.0135 (18)	0.0064 (17)	−0.0018 (13)	0.0008 (14)	−0.0002 (14)
Si1	0.0086 (6)	0.0130 (7)	0.0091 (7)	−0.0004 (5)	−0.0014 (5)	0.0002 (6)
C13	0.016 (3)	0.018 (3)	0.016 (3)	−0.001 (2)	−0.002 (2)	0.005 (2)
C14	0.020 (3)	0.022 (3)	0.010 (3)	−0.001 (2)	0.001 (2)	0.000 (2)
C15	0.015 (2)	0.028 (3)	0.016 (3)	−0.004 (3)	0.002 (2)	0.003 (2)

### Geometric parameters (Å, º)

**Table:**

Br2—C6	1.926 (5)	C8—H8C	0.9800
N1—C5	1.319 (6)	C9—H9A	0.9800
N1—C1	1.475 (6)	C9—H9B	0.9800
N1—C2	1.476 (6)	C9—H9C	0.9800
C1—H1A	0.9800	C10—O1	1.445 (6)
C1—H1B	0.9800	C10—C12	1.515 (7)
C1—H1C	0.9800	C10—C11	1.530 (6)
C2—H2A	0.9800	C11—H11A	0.9800
C2—H2B	0.9800	C11—H11B	0.9800
C2—H2C	0.9800	C11—H11C	0.9800
N2—C5	1.333 (6)	C12—H12A	0.9800
N2—C4	1.470 (6)	C12—H12B	0.9800
N2—C3	1.475 (6)	C12—H12C	0.9800
C3—H3A	0.9800	O1—Si1	1.669 (3)
C3—H3B	0.9800	Si1—C15	1.854 (5)
C3—H3C	0.9800	Si1—C13	1.860 (5)
C4—H4A	0.9800	Si1—C14	1.862 (5)
C4—H4B	0.9800	C13—H13A	0.9800
C4—H4C	0.9800	C13—H13B	0.9800
C5—C6	1.491 (7)	C13—H13C	0.9800
C6—C7	1.327 (7)	C14—H14A	0.9800
C7—N3	1.433 (6)	C14—H14B	0.9800
C7—C10	1.542 (7)	C14—H14C	0.9800
N3—C8	1.458 (6)	C15—H15A	0.9800
N3—C9	1.464 (6)	C15—H15B	0.9800
C8—H8A	0.9800	C15—H15C	0.9800
C8—H8B	0.9800		
			
C5—N1—C1	120.3 (4)	N3—C9—H9B	109.5
C5—N1—C2	124.4 (4)	H9A—C9—H9B	109.5
C1—N1—C2	114.6 (4)	N3—C9—H9C	109.5
N1—C1—H1A	109.5	H9A—C9—H9C	109.5
N1—C1—H1B	109.5	H9B—C9—H9C	109.5
H1A—C1—H1B	109.5	O1—C10—C12	110.4 (4)
N1—C1—H1C	109.5	O1—C10—C11	109.1 (4)
H1A—C1—H1C	109.5	C12—C10—C11	110.9 (4)
H1B—C1—H1C	109.5	O1—C10—C7	108.6 (4)
N1—C2—H2A	109.5	C12—C10—C7	109.5 (4)
N1—C2—H2B	109.5	C11—C10—C7	108.2 (4)
H2A—C2—H2B	109.5	C10—C11—H11A	109.5
N1—C2—H2C	109.5	C10—C11—H11B	109.5
H2A—C2—H2C	109.5	H11A—C11—H11B	109.5
H2B—C2—H2C	109.5	C10—C11—H11C	109.5
C5—N2—C4	122.8 (4)	H11A—C11—H11C	109.5
C5—N2—C3	124.1 (4)	H11B—C11—H11C	109.5
C4—N2—C3	112.7 (4)	C10—C12—H12A	109.5
N2—C3—H3A	109.5	C10—C12—H12B	109.5
N2—C3—H3B	109.5	H12A—C12—H12B	109.5
H3A—C3—H3B	109.5	C10—C12—H12C	109.5
N2—C3—H3C	109.5	H12A—C12—H12C	109.5
H3A—C3—H3C	109.5	H12B—C12—H12C	109.5
H3B—C3—H3C	109.5	C10—O1—Si1	128.7 (3)
N2—C4—H4A	109.5	O1—Si1—C15	106.0 (2)
N2—C4—H4B	109.5	O1—Si1—C13	112.4 (2)
H4A—C4—H4B	109.5	C15—Si1—C13	106.3 (2)
N2—C4—H4C	109.5	O1—Si1—C14	113.5 (2)
H4A—C4—H4C	109.5	C15—Si1—C14	109.4 (2)
H4B—C4—H4C	109.5	C13—Si1—C14	109.0 (2)
N1—C5—N2	123.6 (4)	Si1—C13—H13A	109.5
N1—C5—C6	119.4 (4)	Si1—C13—H13B	109.5
N2—C5—C6	116.6 (4)	H13A—C13—H13B	109.5
C7—C6—C5	129.3 (4)	Si1—C13—H13C	109.5
C7—C6—Br2	121.8 (4)	H13A—C13—H13C	109.5
C5—C6—Br2	108.8 (3)	H13B—C13—H13C	109.5
C6—C7—N3	124.5 (5)	Si1—C14—H14A	109.5
C6—C7—C10	123.9 (4)	Si1—C14—H14B	109.5
N3—C7—C10	111.6 (4)	H14A—C14—H14B	109.5
C7—N3—C8	117.4 (4)	Si1—C14—H14C	109.5
C7—N3—C9	115.8 (4)	H14A—C14—H14C	109.5
C8—N3—C9	113.7 (4)	H14B—C14—H14C	109.5
N3—C8—H8A	109.5	Si1—C15—H15A	109.5
N3—C8—H8B	109.5	Si1—C15—H15B	109.5
H8A—C8—H8B	109.5	H15A—C15—H15B	109.5
N3—C8—H8C	109.5	Si1—C15—H15C	109.5
H8A—C8—H8C	109.5	H15A—C15—H15C	109.5
H8B—C8—H8C	109.5	H15B—C15—H15C	109.5
N3—C9—H9A	109.5		
			
C1—N1—C5—N2	167.2 (5)	C6—C7—N3—C8	77.4 (6)
C2—N1—C5—N2	−23.8 (8)	C10—C7—N3—C8	−105.1 (5)
C1—N1—C5—C6	−20.1 (7)	C6—C7—N3—C9	−61.4 (6)
C2—N1—C5—C6	148.9 (5)	C10—C7—N3—C9	116.1 (5)
C4—N2—C5—N1	161.7 (5)	C6—C7—C10—O1	−7.7 (6)
C3—N2—C5—N1	−25.9 (7)	N3—C7—C10—O1	174.8 (4)
C4—N2—C5—C6	−11.3 (7)	C6—C7—C10—C12	−128.4 (5)
C3—N2—C5—C6	161.1 (4)	N3—C7—C10—C12	54.1 (5)
N1—C5—C6—C7	104.0 (6)	C6—C7—C10—C11	110.6 (5)
N2—C5—C6—C7	−82.7 (6)	N3—C7—C10—C11	−66.9 (5)
N1—C5—C6—Br2	−78.9 (5)	C12—C10—O1—Si1	−40.7 (5)
N2—C5—C6—Br2	94.4 (4)	C11—C10—O1—Si1	81.4 (5)
C5—C6—C7—N3	169.1 (5)	C7—C10—O1—Si1	−160.9 (3)
Br2—C6—C7—N3	−7.7 (7)	C10—O1—Si1—C15	−163.6 (4)
C5—C6—C7—C10	−8.1 (8)	C10—O1—Si1—C13	80.7 (4)
Br2—C6—C7—C10	175.1 (3)	C10—O1—Si1—C14	−43.5 (4)

### Hydrogen-bond geometry (Å, º)

**Table:**

*D*—H···*A*	*D*—H	H···*A*	*D*···*A*	*D*—H···*A*
C3—H3*C*···Br1^i^	0.98	2.81	3.742 (3)	159
C14—H14*B*···Br1^ii^	0.98	2.87	3.790 (3)	156

Symmetry codes: (i) −*x*+3/2, *y*−1/2, *z*; (ii) −*x*+1, −*y*+1, −*z*.
