# Supplementary figures and images for: Crystal structure of 2-bromo-3-di­methyl­amino-N,N,N′,N′,4-penta­methyl-4-(tri­methyl­sil­yloxy)pent-2-eneamidinium bromide
Source: Acta Crystallogr E Crystallogr Commun. 2015 Dec 16;71(Pt 12):o1061–2. doi: 10.1107/S205698901502383X (PMC4719979; doi:10.1107/S205698901502383X)

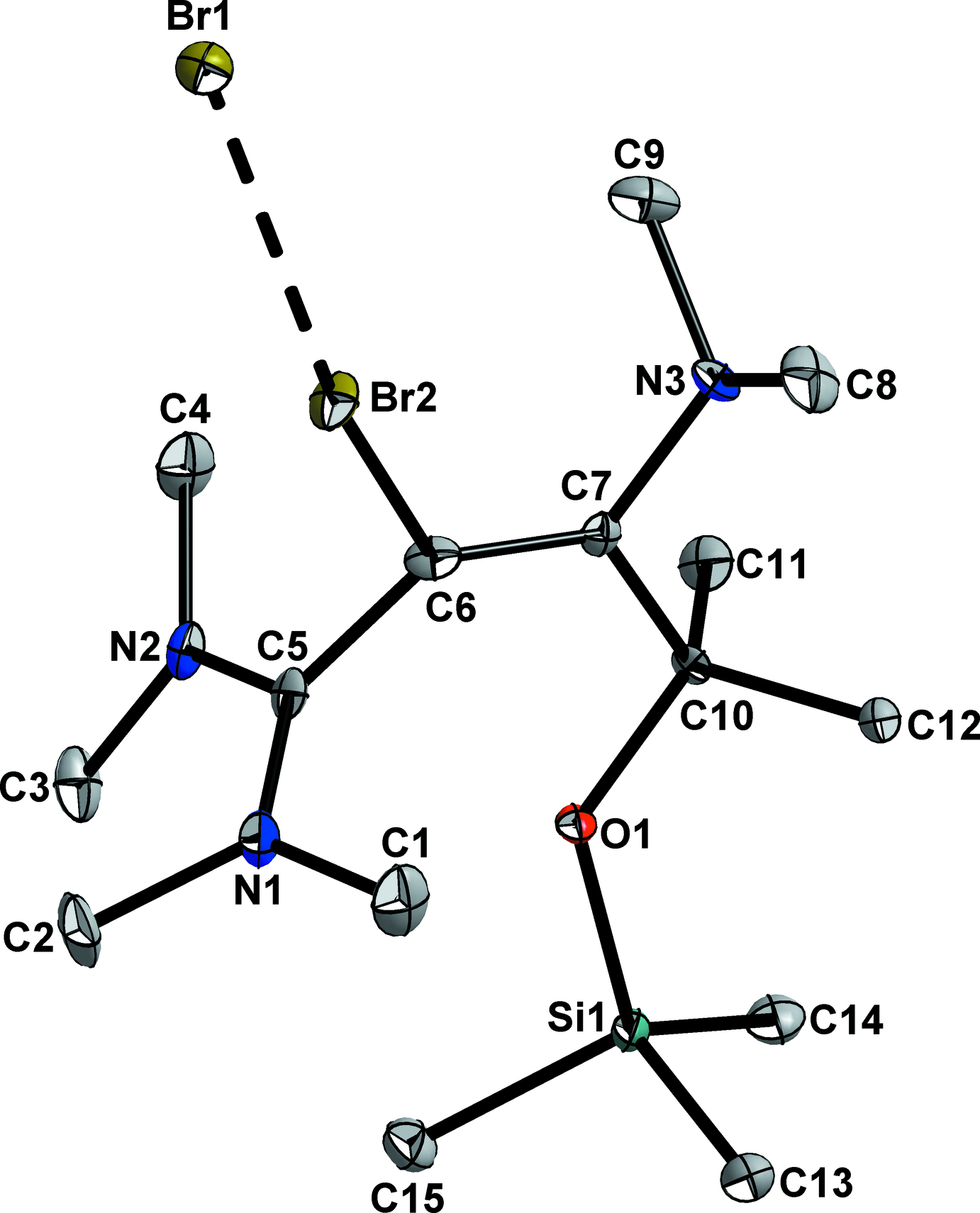

Supplement: Supplementary file 2 [file e-71-o1061-fig1.tif]

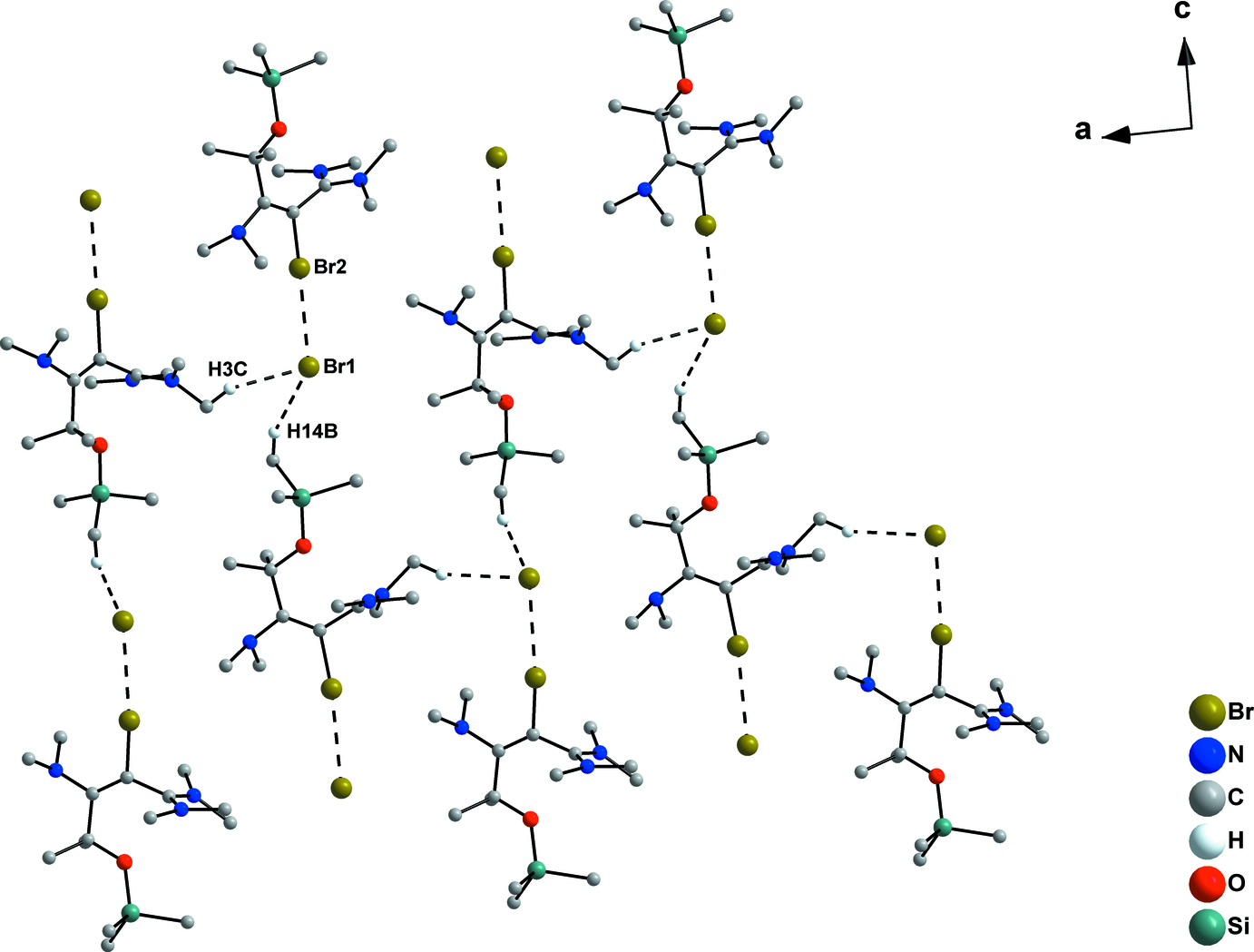

Supplement: Supplementary file 3 [file e-71-o1061-fig2.tif]
